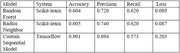# Python‐Based Machine Learning for Three‐Dimensional Model Segmentation and Analysis

**DOI:** 10.1002/alz70855_097048

**Published:** 2025-12-23

**Authors:** Morgan Mekale Smith, Elizabeth Ochoa, Mallory Keating, Margaret E Flanagan, Kevin F. Bieniek

**Affiliations:** ^1^ University of Texas Health Science Center at San Antonio, San Antonio, TX, USA; ^2^ University of Houston, Houston, TX, USA; ^3^ Glenn Biggs Institute for Alzheimer's and Neurodegenerative Diseases, UT Health San Antonio, San Antonio, TX, USA

## Abstract

**Background:**

Pathology and cell loss in neurodegenerative diseases results in brain atrophy of specific neuroanatomical structures. Atrophy can be quantitatively assessed using neuroimaging techniques, such as magnetic resonance imaging (MRI), but these methods can be costly. We seek to use three‐dimensional (3D) modeling technologies and machine learning (ML) to permit visualization and quantitative analysis approaches, including model segmentation.

**Method:**

Python and Blender were utilized for neuroanatomical analysis and 3D model manipulation. Structured‐light scanning was used to generate 3D models of postmortem brain tissue, which were imported as .obj files and simplified via mesh decimation in Python. The decimated models were manually segmented in Blender based on identifiable gyri and sulci, then exported as .csv files for a Python‐based ML pipeline. Model performance was evaluated using accuracy, precision, recall, F1‐score, and hamming loss.

**Result:**

Our methods demonstrated that in our scikit‐learn pipeline, random forest and radius neighbor classifiers generated predictions with the highest accuracy, average precision, and recall scores when assigning labels to unannotated data compared to 11 scikit‐learn ML models. In addition, these algorithms demonstrated the lowest hamming loss scores. Our custom sequential Tensorflow model outperformed these models, demonstrating improved accuracy scores, lower precision and recall, and slightly elevated binary cross entropy loss measurements. Our pipelines provide evidence to suggest that predictive modeling can be used to segment multiple 3D models using multi‐label classification techniques with accuracy and precision scores of 60‐90% (Figure 1).

**Conclusion:**

Our findings demonstrate the potential of ML techniques to identify neuroanatomical sub‐regions and measure atrophy from 3D brain models. Future validation with other neuroimaging modalities is essential to ensure consistency in surface mesh segmentation and cortical atrophy measurements. Addressing class imbalance and fine‐tuning hyperparameters can further enhance evaluation metrics. Integrating annotated 2D images with segmented 3D models using ML techniques may yield data comparable to MRI‐based cortical atrophy measurements. This approach, supported by prior work (Jang et al., 2022), could combine surface and volumetric insights, offering valuable tools for neuropathology and biomedical engineering in practice, research, and education.